# Neuropsychological Profile of Specific Executive Dysfunctions in Patients with Deficit and Non-deficit Schizophrenia

**DOI:** 10.3389/fpsyg.2017.01459

**Published:** 2017-08-30

**Authors:** Ernest Tyburski, Justyna Pełka-Wysiecka, Monika Mak, Agnieszka Samochowiec, Przemysław Bieńkowski, Jerzy Samochowiec

**Affiliations:** ^1^Department of Clinical Psychology, Institute of Psychology, University of Szczecin Szczecin, Poland; ^2^Department of Psychiatry, Pomeranian Medical University Szczecin, Poland; ^3^Independent Clinical Psychology Unit, Department of Psychiatry, Pomeranian Medical University Szczecin, Poland; ^4^Department of Psychiatry, Medical University of Warsaw Warsaw, Poland

**Keywords:** executive functions, concept formation, verbal cognitive flexibility, non-verbal cognitive flexibility, deficit schizophrenia

## Abstract

**Objectives:** Although it has been shown that there are more profound deficits present in deficit schizophrenia (DS) patients than in non-deficit schizophrenia (NDS) patients, there still remain some matters requiring further investigation. In this context, we formulated three research aims: (1) to compare executive functions between the investigated groups, (2) to determine the relationship between particular aspects of executive functions within the groups, and (3) to draw up a neuropsychological profile for executive functions.

**Methods:** The study involved 148 schizophrenia patients divided into two groups on the basis of the Schedule for the Deficit Syndrome: DS (*n* = 70) and NDS (*n* = 78). Patients were matched for sex, age, years of education, and overall cognitive functioning. For assessing executive functions we used the Wisconsin Card Sorting Test (WCST), the Trail Making Test (TMT), the Phonemic Verbal Fluency Test (VFT P), the Stroop Color and Word Test (SCWT), and the Go/No Go task (GNG).

**Results:** Deficit schizophrenia patients scored lower on the WCST and TMT (relative flexibility) than did the NDS patients. There were no inter-group differences in the VFT P, SCWT (relative inhibition), or GNG. There were significant correlations between WCST and TMT scores in both groups. The general neuropsychological profiles were similar in both groups.

**Conclusion:** Deficit schizophrenia patients exhibited slightly greater interference with concept formation and non-verbal cognitive flexibility. Therefore, such problems may be specific to this particular type of schizophrenia. These results may be useful for the development of neuropsychological diagnostic methods for patients with schizophrenia.

## Introduction

There is an ongoing discussion about whether different types of schizophrenia are associated with specific types of executive dysfunction (Brazo et al., 2002; Simon et al., 2009; Fioravanti et al., 2012; Hegde et al., 2013; Ventura et al., 2013). The heterogeneity of schizophrenia symptoms has led to a distinction between different clinical syndromes within a single disease. The term ‘deficit schizophrenia’ was first suggested by Carpenter et al. (1988) as a type of schizophrenia with dominant negative symptoms persisting for a long time. Among these are persistent and primary negative symptoms such as social withdrawal, poverty of speech, limited content of verbal expression, apathy, and blunting of affect (Strauss et al., 2010). Longitudinal analyses show that these symptoms are stable over time (Tek et al., 2001; Chemerinski et al., 2006; Strauss et al., 2010). There are numerous reports confirming the validity of deficit schizophrenia (DS) diagnoses (Tek et al., 2001; Arango et al., 2004; Messias et al., 2004; Dickerson et al., 2006; Cohen et al., 2007; Galderisi et al., 2008; Kirkpatrick and Galderisi, 2008; Pełka-Wysiecka et al., 2013). However, apart from negative/deficit symptoms, the basic symptomatic dimensions in schizophrenia include also reality distortion and disorganization (Liddle et al., 1992; Schröder et al., 1992). The occurrence of the two latter types of symptoms may also be associated with executive function impairments.

The construct of executive functions has enabled a more insightful understanding of the self-regulatory processes responsible for the management of one’s thoughts, emotions, and behavior (Alvarez and Emory, 2006; Jurado and Rosselli, 2007; Diamond, 2013). In clinical neuropsychology, it has been assumed that they form a superordinate system which allows the implementation of purposeful action, and involves four domains: volition, planning, purposive action, and effective performance (Lezak, 1995; Jodzio, 2008). Many clinical and experimental studies have confirmed that these functions are carried out by a complex central executive network which includes a variety of brain structures, the most important of which are the prefrontal cortex, the anterior cingulate cortex, the subcortical nuclei, and the cerebellum (Stuss, 2011; Niendam et al., 2012; Yuan and Raz, 2014; Mak et al., 2016). Many studies suggest the presence of greater structural and functional disorders of the brain in DS patients than in their NDS counterparts (Liddle et al., 1992; Tamminga et al., 1992; DeQuardo et al., 1998; Heckers et al., 1999; Lahti et al., 2001). Based on these studies, Buchanan et al. (1994) and Kirkpatrick et al. (2001) asserted that malfunctioning of the loop created by the prefrontal cortex, the inferior parietal cortices, and the thalamus is implicated in the pathophysiology and executive dysfunctions of the deficit syndrome in schizophrenia.

As can be seen in **Table 1**, neuropsychological analyses of the executive functioning of patients with DS and non-deficit schizophrenia (NDS) yield somewhat inconsistent results. Polgár et al. (2010) showed that patients with DS achieved lower scores than those with NDS in specific measures of the Wisconsin Card Sorting Test (WCST). In addition, a factor analysis was performed, showing that there are at least two factors relating to mental processes engaged in this test. The first is concept formation and flexibility, and it includes, inter alia, Perseverative Responses (PR), and Perseverative Errors (PE). The second is unsuccessful problem-solving with an ineffective hypothesis-testing strategy and includes Non-perseverative Errors (NPE). Analysis of the results showed that only some DS patients obtained lower PE scores than did those with NDS (**Table 1**). Other reports found no inter-group differences (or differences in PR score, see **Table 1**). These particular scores are not considered at all in some papers. Furthermore, Wang et al. (2008) and Vogel et al. (2013) report some contradictory findings, as their subjects differed in terms of PE scores, but not PR scores. NPE scores were only considered in four papers, and only Réthelyi et al. (2012) found that patients with DS had lower scores than those with NDS. A review of research which used the Trail Making Test (TMT, version B) revealed that, in some papers, patients with DS scored lower than NDS patients. Unfortunately, only two papers reported patient scores for absolute non-verbal cognitive flexibility [time B – A], some independent of the speed of information processing (TMT AF, Chan et al., 2015). In the study of Wang et al. (2008), patients with DS obtained lower scores than those with NDS, while Galderisi et al. (2002) did not report any inter-group differences. A meta-analysis of research which used the Phonemic Verbal Fluency Test (VFT P) to measure verbal cognitive flexibility showed that DS patients scored lower than NDS patients in three studies, while in five others there were no reports of any inter-group differences. A review of studies which used the Stroop Color and Word Test (SCWT) showed that only in the study by Réthelyi et al. (2012) did DS patients score lower than NDS patients in the task of reading the names of colors printed in a color different (incongruent) to that denoted by the name. Cohen et al. (2007) found no inter-group differences. Buchanan et al. (1994) was the only study in which the interference index was applied, where reaction time was controlled for the congruent trial. The authors showed that DS patients exhibited higher (worse) scores than did the NDS patients. We could not find any available research on DS patients performing the Go/No Go task (GNG).

**Table 1:** Survey of studies on PubMed which test executive functions in deficit schizophrenia (DS) and non-deficit schizophrenia (NDS) patients, and normal controls (CON).

Authors	Number of DS/NDS/CON	WCST			TMT		VFT P	SCWT	
					
		WCST PR/%	WCST PE/%	WCST NPE/%	TMA B	TMT AF		SCWT I	SCWT AI
Buchanan et al., 1994	18/21/30^a,b,c^	ns	ni	ni	<0.05	ni	ns	ni	ni
Bryson et al., 2001	33/57/none^a,b,c^	ni	<0.05	ni	ni	ni	ni	ni	ni
Galderisi et al., 2002	58/54/26^a,b,c^	ni	ns	ni	ni	ns	ni	ni	ni
Horan and Blanchard, 2003	15/30/41^a,c^	ni	<0.05	ni	ni	ni	ni	ni	ni
Tiryaki et al., 2003	19/43/none^a,b,c^	ni	ni	ni	ns	ni	ns	ns	ni
Delamillieure et al., 2004	5/17/21^a,b,c^	ni	ni	ni	ni	ni	ni	ns	ni
Cohen et al., 2007	20/25/25^a,b,c^	ni	ns	ni	ns	ni	ns	ni	ns
Cascella et al., 2008	26/79/316^a,b,c^	ni	ns	ni	ns	ni	ns	ni	ni
Polgár et al., 2008	27/45/30^a,b,c^	ni	<0.05	ni	<0.05	ni	<0.05	ni	ni
Wang et al., 2008	30/93/103^a,b,c^	ns	<0.05	ns	<0.01	<0.01	ni	ni	ni
Polgár et al., 2010	154/121/130^a,b,c^	<0.05	<0.05	<0.05	ni	ni	ni	ni	ni
Réthelyi et al., 2012	143/123/none^a,b^	ni	<0.001	<0.001	<0.001	ni	<0.001	<0.001	ni
Vogel et al., 2013	15/52/51^a,b,c^	ns	<0.01	ns	ni	ni	ni	ni	ni
Csukly et al., 2014	30/28/29^a,b,c^	ni	<0.001	ni	ni	ni	ni	ns	ni
Scala et al., 2014	15/40/55^a,b,c,d^	ns	ns	ns	ni	ni	ns	ni	ni
Yu et al., 2015	40/57/52^b^	ni	ni	ni	<0.001	ni	<0.05	ns	ni


*ni, no information; ns, no significant; WCST, Wisconsin Card Sorting Test; PR, Perseverative Responses; PE, Perseverative Errors; NPE, Non-perseverative Errors; TMT, Trail Making Test; B, time; AF, Absolute Flexibility [time B – A]; VFT P, Phonemic Verbal Fluency Test; SCWT, Stroop Color and Word Test: I, Incongruent; AI, Absolute Inhibition [time incongruent – congruent].*

*^a^Deficit schizophrenia and non-deficit schizophrenia and normal controls matched for gender.*

*^b^Age.*

*^c^Years of education.*

*^d^Premorbid intelligence quotient.*

Furthermore, the specific relationship between the particular aspects of DS and NDS may prove important for understanding the nature of executive functions in DS/NDS patients, as demonstrated in research conducted on healthy persons (Miyake et al., 2000) and older subjects (McCabe et al., 2010; Brown et al., 2012). Unfortunately, such relationships have been very rarely examined in this group of patients. Only Yu et al. (2015) managed to demonstrate a significant correlation between scores on the TMT and VFT P in patients with DS. It may also be important to identify which aspects of executive function are most impaired in patients with DS and NDS. This is made possible by a profile analysis of neuropsychological function (Lezak et al., 2004; Voglmaier et al., 2005). Brazo et al. (2002) found the greatest disturbance in patients with DS in areas of concept formation (Modified Card Sorting Test, MCST) and verbal cognitive flexibility (VFT P), and their non-verbal cognitive flexibility (TMT) and cognitive inhibition (SCWT) were least affected. The aforementioned functions remained on a similar level in NDS patients. In turn, Cascella et al. (2008) demonstrated that DS patients exhibit the greatest difficulty with speed of information processing and verbal cognitive flexibility (VFT P), and they tend to do slightly better in concept formation (MCST), with a similar profile observed in both DS and NDS patients. However, Réthelyi et al. (2012) and Yu et al. (2015) showed that patients with DS and NDS have greater problems with regards to non-verbal flexibility (TMT), than with verbal cognitive flexibility (VFT P).

As can be seen in the above results, there are still a few unresolved issues concerning executive function in patients with DS. First of all, the precise nature of executive dysfunction in this group of patients has not been established. Secondly, it is not clear what is the relationship between various aspects of the executive function in those patients or whether there exists any at all. Also, it is not fully known which domains of the described processes suffer the greatest impairment within the group. Therefore, both the inconclusiveness of findings and the importance of executive functions for the performance of complex actions have led to formulation of three research aims: (1) to compare executive function performance between the investigated groups, (2) to determine the relationship between the particular aspects of executive functions within the groups, and (3) to draw up a neuropsychological profile for executive functions which takes into account the diversity of the different aspects of these processes.

## Materials and Methods

### Participants

The patient group consisted of 148 right-handed Caucasians (74 female and 74 male) who had been diagnosed with schizophrenia according to ICD-10 (World Health Organization [WHO], 1992) for a minimum of 18 months. Patient interviews were done by properly licensed psychiatrists. Among the inclusion criteria were the ability to understand the research procedure, being aged between 20 and 60, and having given informed consent. Exclusion criteria were other mental diseases, neurological diseases, dementia, a history of traumatic brain injury, and severe diseases of the parenchymal organs, a history of alcohol or drug misuse, or intellectual disability. With the construction of the study in mind, patients who exhibited clear symptoms of disorganization were also excluded. The patients were recruited from inpatient psychiatric wards, psychiatric daycare wards, and outpatient clinics in the Western Pomerania district of Poland. All subjects were fully informed about the aims and the protocol of the study and all gave written informed consent. The protocol was approved by the local bioethics committee.

### Measures

#### Clinical Assessment

The presence of psychopathological symptoms was assessed using the Positive and Negative Syndrome Scale (PANSS, Kay et al., 1987), and the Clinical Global Impression – Schizophrenia scale (CGI-SCH, Haro et al., 2003), which assessed four groups of symptoms (positive, negative, depressive, and cognitive) during a psychiatric examination. To describe the severity and type of deficit symptoms, we used a Polish translation of the Schedule for the Deficit Syndrome (SDS, Kirkpatrick et al., 1989). DS was diagnosed by the presence of the following negative symptoms: restricted affect, diminished emotional range, poverty of speech, curbing of interests, diminished sense of purpose, and diminished social drive. All the above symptoms had to be primary, i.e., not caused by positive symptoms such as depression, cognitive dysfunction, psychopharmacotherapy, or poor general health, and had to have been present for the preceding 12 months.

The patients were in symptomatic remission, not acute psychosis. All subjects were treated according to the guidelines for the psychopharmacological treatment of schizophrenia. In both groups the patients received typical (perazine, zuclopenthixol, haloperidol) or atypical (risperidone, olanzapine, clozapine, quetiapine, aripiprazole, amisulpride) antipsychotics. The DS and NDS groups did not differ in terms of type of neuroleptics used.

#### Neuropsychological Assessment

In this study we used the WCST in its original computerized form (Heaton et al., 1993; Jaworowska, 2002). Based on data collected by Polgár et al. (2010), we decided to measure concept formation using two scores: PE and PR, and to assess problem-solving using NPE. The subject’s task was to discover the rule that is currently in place (color, shape or number) and answer by pressing the right key on the keyboard, from 1 to 4 based on the feedback (correct or incorrect) displayed on a 15″ screen. Before the test, each participant received instructions from a psychologist. For the assessment of non-verbal cognitive flexibility, we used the TMT (Reitan, 1958). However, bearing in mind that DS and NDS patients’ speed of information processing is generally slower (Morrens et al., 2007), we decided to use the Relative Flexibility indicator (TMT), applying the formula: [(time B - A/time B) × 100] (Stuss et al., 2001; Perianez et al., 2007). In TMT A, subjects had to connect 25 circles containing numbers from 1 to 25, which were irregularly placed on a white, A4 sheet, with a continuous line, as quickly as possible. TMT B consisted of connecting circles, going by turns from number to letter, while preserving the order of numbers and following alphabet (from 1 to A, from A to 2, etc.), finishing at number “13” and the letter “L.” A practice trial was done before each task so that the investigator could be sure that the patient understood the instructions. Instructions were provided verbally by the investigator (psychologist) both before the practice task and the actual task. In turn, to assess verbal cognitive flexibility, we administered the VFT P (Lezak, 1995; Tyburski et al., 2015). Each individual was asked to list as many words as they can, as fast as possible, according to the given criterion (words beginning with k or p). The time for completing each trial was 60 s. The researcher wrote down each word on an answer sheet. Since it has been demonstrated that the number of correctly spoken words strongly correlates with the number of word switches, this indicator was considered to be a good measure of verbal cognitive flexibility (Ross, 2003). We also assessed cognitive inhibition (dominant verbal response) by means of the SCWT. However, because patients with DS exhibit slowing of information processing (Morrens et al., 2007), we decided to use the Relative Inhibition Indicator (SCWT RI) in the formula: [(time incongruent - congruent/time congruent) × 100] (Denney and Lynch, 2009). In the first task, the subject had to read aloud as fast as possible the names of colors printed in a black font on a white A4 sheet. In the second task, the subject had to name the colors of words printed in a colored font, where the font color was incongruent with the word’s meaning (e.g., the word “green” printed in red). Instructions were provided verbally each time by the investigator (psychologist) before the task. The computer version of the GNG was also used and motor inhibition was measured with the number of No Go type errors (Strauss et al., 2006; Wright et al., 2014). The subject’s task was to press the spacebar on the keyboard when a green square appeared on the computer screen (15″), and to refrain from pressing the spacebar when a blue square appeared on the screen. Instructions were presented on the computer screen before the task.

### Procedure

At their first appointment, all patients were examined by one of four psychiatrists who carried out a structured interview and assessment based on clinical scales (each patient was evaluated using the PANSS, CGI-SCH, and SDS). The psychiatrists were members of the research team and had been trained in the research procedure, including the use of the psychiatric scales. The next appointment involved neuropsychological assessment, carried out by one of three trained psychologists. All patients were examined with the same neuropsychological battery. Administration of each tool was preceded by the standard instructions.

### Statistical Analysis

Statistical analysis of the results was done using the IBM SPSS 21 Statistical package. Continuous variables were presented as means (*M*) and standard deviations (*SD*) or standard errors (*SE*). The normality of the distribution was tested with the Shapiro–Wilk test. Before any analyses were conducted, square root transformation was used to transform the raw results of variables which were not normally distributed. Then selected scores were transformed into unitarized results using the formula x_u_ = [(x_i_ - min)/(max - min) × 100] (ranges from 0 to 100, the higher the score, the more difficult the task). To check for differences between the groups, the non-parametric Mann–Whitney *U*-test (for demographic and clinical variables) or parametric Student’s *t*-tests were used (for neuropsychological variables). The Wendt *r*_U_ rank-biserial correlation method (Wendt, 1972; Rosenthal and Rubin, 2003) was used to determine the magnitude of effect size measures for the non-parametric tests and Cohen’s *d* or η^2^ effect size (Cohen, 1992) was used to determine the magnitude of effect size measures for the parametric test and analysis of variance (ANOVA). For multiple comparisons the Bonferroni correction was used. To assess the strength of the relationship between different aspects of executive functioning, Pearson’s *r* correlation coefficient was used. To draw up the executive function profile and compare the results from different neuropsychological tests, we used a repeated measures/mixed model ANOVA. We assumed the group type (DS or NDS) as the inter-object factor, and the aspect of executive function (the type of measure) as an intra-object 7-level factor scale.

## Results

### Subjects’ Characteristics

The patients’ socio-demographic and clinical characteristics are shown in **Table 2**. Neither investigated group differed in terms of number of years of education, gender, length of time since diagnosis, level of general mental functioning (assessed with MMSE), or number of hospitalizations at psychiatric wards. DS patients had higher scores than non-deficit patients on all PANSS (*p* < 0.001) and SGI-SCH subscales (*p* < 0.001). The effect size (*r*_U_) was found to be 0.25–0.74, i.e., a small to large effect size.

**Table 2:** Demographic and clinical characteristics of deficit schizophrenia (DS) and non-deficit schizophrenia (NDS) patients.

	DS (*n* = 70)	NDS (*n* = 78)	*Z*/χ^2^	*p*
Age (years): *M* (*SD*)	40.94 (9.95)	39.17 (11.24)	-1.07^a^	0.284
Education (years): *M* (*SD*)	12.23 (2.55)	12.88 (2.77)	-1.42^a^	0.156
Sex: male/female	38/32	36/42	0.68^b^	0.410
Relationship: yes/no	22/48	25/53	0.00^b^	1.000
Duration of illness (years): *M* (*SD*)	13.79 (7.08)	12.14 (8.10)	-1.82^a^	0.068
Number of hospitalizations: *M* (*SD*)	7.54 (6.18)	6.55 (5.61)	-1.10^a^	0.270
PANSS P: *M* (*SD*)	6.11 (4.35)	4.86 (5.41)	-2.58^a^	0.010
PANSS N: *M* (*SD*)	15.67 (5.97)	6.55 (5.47)	-7.80^a^	0.000
PANSS G: *M* (*SD*)	18.19 (8.75)	9.99 (8.45)	-5.52^a^	0.000
CGI-SCH: *M* (*SD*)	3.17 (1.09)	1.97 (0.91)	-6.38^a^	0.000
MMSE: *M* (*SD*)	28.23 (1.48)	28.46 (1.74)	-1.50^a^	0.137


*PANSS, Positive and Negative Syndrome Scale; P, Positive; N, Negative; G, General; CGI-SCH, Clinical Global Impression – Schizophrenia; MMSE, Mini Mental State Examination.*

*^a^Mann–Whitney *U*-test.*

*^b^Chi-square test.*

### Performance in Specific Aspects of Executive Functions

As shown in **Table 3**, DS patients scored lower in concept formation (WCST PR: *p* < 0.05; WCST PE: *p* < 0.05) and non-verbal cognitive flexibility (TMT RF: *p* < 0.05) in comparison to NDS patients. The effect size (*d*) of executive dysfunctions in WCST and TMT was found to be 0.38–0.39, indicating a small effect size. No differences were observed in verbal cognitive flexibility (VFT P) and cognitive (SCWT RI) or motor inhibition (GNG).

**Table 3:** Comparison of raw scores of executive performance for deficit schizophrenia (DS) versus non-deficit schizophrenia (NDS) patients.

Aspects of executive functions	Tests and index	DS (*n* = 70) *M* (*SD*)	NDS (*n* = 78) *M* (*SD*)	*t*	*p*	*d*	Effect size
Concept formation	WCST PR	37.31 (23.58)	28.48 (22.26)	2.35	0.020	0.39	Small
	WCST PE	39.39 (24.16)	30.46 (22.66)	2.32	0.022	0.38	Small
Problem-solving	WCST NPE	37.90 (20.99)	33.46 (16.17)	1.43	0.154	–	None
Non-verbal cognitive flexibility	TMT RF	50.48 (18.41)	43.72 (17.83)	2.27	0.025	0.38	Small
Verbal cognitive flexibility	VFT P	60.88 (19.98)	63.16 (17.42)	-0.74	0.461	–	None
Cognitive inhibition	SCWT RI	44.57 (17.18)	42.28 (13.91)	-0.88	0.382	–	None
Motor inhibition	GNG NGE	29.56 (24.83)	30.02 (22.14)	-0.12	0.905	–	None


*WCST, Wisconsin Card Sorting Test; PR, Perseverative Responses; PE, Perseverative Errors; NPE, Non-perseverative Errors; TMT, Trail Making Test: RF, Relative Flexibility *[(time B-A/time B-A/time A)* × *100]*; VFT P, Phonemic Verbal Fluency Test; SCWT, Stroop Color and Word Test; RI, Relative Inhibition *[(time incongruent-congruent/time congruent)* × *100]*; GNG NGE, Go/No Go Task, No Go Errors.*

### Associations between Particular Aspects of Executive Functions

As can be seen in **Table 4**, there was a strong positive correlation between the two measures relating to concept formation (WCST PR and PE) in both groups, as well as a weak positive correlation between measures relating to concept formation (WCST PR and PE) and problem-solving (WCST NPE), and a small positive correlation between measures of concept formation (WCST PR and PE) and non-verbal cognitive flexibility (VFT P), as well as problem-solving and cognitive inhibition (SCWT RI). In addition, DS patients showed a slight positive correlation between measures relating to concept formation (WCST PR and PE) and cognitive inhibition (SCWT IR), as well as non-verbal (TMT RI) and verbal cognitive flexibility (VFT P). In turn, in patients with NDS, there was a positive correlation between measures relating to concept formation (WCST PR and PE), problem-solving (WCST NPE), and verbal cognitive flexibility (VFT P).

**Table 4:** Correlation (Pearsons’) between particular aspects of executive function for deficits schizophrenia (DS) and non-deficits schizophrenia (NDS) patients.

		Group	Concept formation	Problem-solving	Non-verbal cognitive flexibility	Verbal cognitive flexibility	Cognitive inhibition	Motor inhibition
		
			WCST PE	WCST NPE	TMT RF	VFT P	SCWT RI	GNG NGE
Concept formation	WCST PR	DS	0.99**	0.26**	0.34**	0.18	0.25*	0.06
		NDS	0.99**	0.40**	0.28*	0.36**	0.17	0.09
	WCST PE	DS		0.31**	0.35**	0.17	0.25*	0.06
		NDS		0.44**	0.29*	0.39**	0.17	0.10
Problem-solving	WCST NPE	DS			0.24*	0.09	0.25*	0.17
		NDS			-0.02	0.42**	0.28*	0.09
Non-verbal cognitive flexibility	TMT FR	DS				0.27*	0.10	0.17
		NDS				0.02	0.18	-0.04
Verbal cognitive flexibility	VFT P	DS					-0.02	0.06
		NDS					0.18	0.08
Cognitive inhibition	SCWT RI	DS						0.22
		NDS						*0.01*


*WCST, Wisconsin Card Sorting Test; PR, Perseverative Responses; PE, Perseverative Errors; NPE, Non-perseverative Errors; TMT, Trail Making Test; RF, Relative Flexibility *[(time B-A/time A)* × *100]*; VFT P, Phonemic Verbal Fluency Test; SCWT, Stroop Color-Word Test; RI, Relative Inhibition *[(time incongruent- congruent/time congruent)* × *100]*; GNG NGE, Go/No Go Task, No Go Errors. ^∗^*p* < 0.05, ^∗∗^*p* < 0.01.*

### Neuropsychological Profile of Executive Functions

**FIGURE 1:**
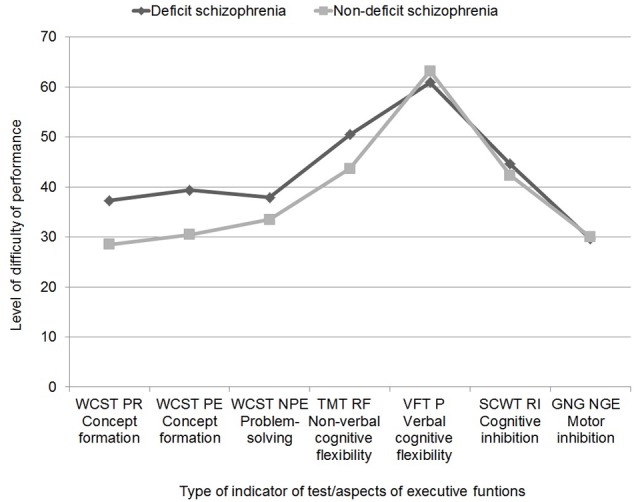
Neuropsychological profile of executive functions in patients with deficit and non-deficit schizophrenia (abbreviations in note in **Table 3**).

**Figure 1** shows the profile of executive functions for both patient groups. ANOVA with repeated measures/mixed model showed significant differences between the different aspects of executive function in both patient groups [*F*(6,608) = 57.41; *p* = 0.000; η^2^ = 2.82]. There was no statistically significant interaction effect between group type and the nature of the executive domain [*F*(6,6.08) = 2.28; *p* = 0.057; η^2^ = 0.02]. Patients with DS (*M* = 42.87; *SE* = 1.43) had higher general scores than patients with NDS, which indicates more severe problems in terms of executive function [*F*(1,146) = 4.30; *p* = 0.040; η^2^ = 0.03]. Pairwise comparison showed that patients with DS scored highest, indicating their greatest difficulties, in the VFT P (*M* = 60.88; *SE* = 2.23), and scored lowest in the GNG (*M* = 29.56; *SE* = 2.80), WCST NPE (*M* = 37.90; *SE* = 2.25), WCST PR (*M* = 37.31; *SE* = 2.74) and WCST EP (*M* = 39.39; *SE* = 2.80). It was similar in patients with NDS – the greatest problems occurred in the performance of VFT P (*M* = 63.16; *SE* = 2.11), and the least problematic were the WCST PR (*M* = 28.48; *SE* = 2.59), GNG (*M* = 30.02; *SE* = 2.66), WCST EP (*M* = 30.46; *SE* = 2.6), and WCST NPE (*M* = 33.46; *SE* = 2.13). In addition, patients with DS had similar results in TMT (*M* = 50.48; *SE* = 2.16) and SCWT RI (*M* = 44.57; *SE* = 1.90), which still differed significantly from the results obtained in the other measures. Patients with NDS also had similar results in TMT RF (*M* = 43.72; *SE* = 2.05) and SCWT RI (*M* = 42.28; *SE* = 1.80), which were also significantly different from the results in the other factors.

## Discussion

The results partially confirmed the first hypothesis. It was found that DS patients had lower levels of concept formation than did patients with NDS. Other researchers report similar findings (**Table 1**). However, in most studies there were only differences in the WCST in the PE score. Only Polgár et al. (2010) report that patients with DS both gave more PR and committed more PE than did NDS patients. Therefore, patients with DS are more likely to have diminished ability to use positive and negative feedback in the learning process and to react optimally to new situations. However, differences in the performance of this test between patients from the two groups could be due to decreased working memory efficiency (working memory is important for holding information in temporary storage, manipulating it, and using it to guide subsequent behavior), which has been noted by, e.g., Park and Gooding (2014). In addition, we have demonstrated that patients with DS have lower levels of non-verbal cognitive flexibility than do NDS patients. However, it was difficult to relate our results to the findings of other researchers, as they did not assess patients’ performance on the TMT (Relative Flexibility Indicator). Wang et al. (2008) found a significant difference between DS/NDS patients regarding their scores on the Absolute Flexibility task, but Galderisi et al. (2002) did not report such a difference. In some studies (**Table 1**), patients with DS had longer response times in this task (part B), but these results should be interpreted with great caution, as there is a strong dependence between this measure and speed of processing information.

There were no inter-group differences in terms of verbal cognitive flexibility, or cognitive or motor inhibition. Admittedly, there are several studies in which patients with DS got lower results in the VFT P (Polgár et al., 2008; Réthelyi et al., 2012; Yu et al., 2015), but other researchers report no inter-group differences (**Table 1**). To interpret these results it may be important to note that the ability to generate words is of a complex nature and requires the use of many mental processes, not only set shifting, but also language competence, psychomotor speed, as well as episodic, semantic, and working memory (Szepietowska and Gawda, 2011). Furthermore, its neural correlates include various cooperating brain regions (Amunts et al., 2004). Therefore, the extent to which this task may be useful for differentiating between deficit and NDS remains a matter for further discussion. The SCWT has not been used with Relative Inhibition in previous research. Though, in the work of Buchanan et al. (1994), patients with DS obtained lower scores on the interference index, which was modified using statistical control of the reaction time in the congruent variant, than did patients with NDS. In addition, only the work by Réthelyi et al. (2012) found that patients with DS had longer response times in the incongruent variant of this task than patients with NDS, but these results may reflect a greater slowing of information processing, rather than large deficits in cognitive inhibition (Knowles et al., 2010). It was also observed that DS and NDS patients obtained similar results in motor response, based on their performance of the GNG. The groups did not differ in terms of inhibiting reactions to irrelevant stimuli (No Go). The existence of any larger deficits in patients with DS than those with NDS in the area of cognitive and motor inhibition requires further research, especially in the context of the assessment of brain activity using functional neuroimaging techniques (Egner and Hirsch, 2005; Aron et al., 2007; Gorfein and MacLeod, 2007; Yücel et al., 2014).

A partial confirmation of the second hypothesis was possible, as we have shown the presence of a relationship between certain aspects of executive function, both in patients with DS and NDS. There were, however, some discrepancies between the patient groups. In both groups there were associations between concept formation, problem-solving, and non-verbal cognitive flexibility. Only in patients with DS were there links between concept formation and cognitive inhibition. In turn, significant correlations between concept formation, problem-solving, and verbal cognitive flexibility were only present in patients with NDS. However, it was difficult to relate these results to the findings of other authors, as the relationship between various executive domains in patients with DS and NDS has not been studied very deeply. Admittedly, Yu et al. (2015) reported that there is an important correlation between performance on the TMT and VFT P in patients with DS. A similar relationship was observed in this study, since there was an association between the TMT Relative Flexibility Indicator and the VFT P.

The third hypothesis was confirmed, as we have demonstrated the presence of significant variation in terms of levels of the individual aspects of executive function in patients with DS and NDS. It was found that, in both groups, patients were weaker in the area of verbal cognitive flexibility than in other executive domains. In addition, patients of both groups performed at the same level in terms of concept formation, problem-solving, and motor inhibition. In turn, non-verbal cognitive flexibility and cognitive inhibition remained at a higher level than verbal cognitive flexibility, but still proved significantly more difficult than the rest of the executive domains. The fact that the executive function profiles in both groups were similar was shown by the small effect size (0.03) of differences in the comparison of overall scores in ANOVA, which means that the analysis explained only 3% of the variation of the general results of the two groups. Our results were consistent with the results obtained by Brazo et al. (2002) and Cascella et al. (2008). Réthelyi et al. (2012) and Yu et al. (2015) reported slightly different results – finding that DS patients exhibited greater difficulties with non-verbal than with verbal cognitive flexibility. The obtained results were partially in line with the results of Chen et al. (2014). They found modest differences between the neuropsychological profiles of first-episode drug naive patients with DS and NDS, as well as between medicated patients with DS and NDS. However, only in the case of the first-episode drug naive patients were differences found between particular cognitive domains – i.e., patients with DS scored lower than those with NDS in terms of speed of processing and attention. However, it was difficult to directly compare the results presented in this paper to those of Chen et al. (2014) because the latter authors used different measurement tools (i.e., the CogState battery) for evaluating cognitive functions.

## Conclusion

The results in this paper are in line with other research and require further empirical validation. An important strength of this study was the use of a neuropsychological test battery for assessing various aspects of executive function in a large patient group. With this data it was possible to consider a broader diagnostic context, which could inform the work of therapeutic teams (Mak et al., 2013). In particular, the ability to detect deficit patients early on in the course of their disease and identify specific executive domains which are impaired may facilitate the implementation of rehabilitation activities, which can help patients function in society (Semkovska et al., 2004; Zipursky, 2014). One limitation of this study would be the lack of control group (e.g., healthy subjects). However, the main goal was to examine the differences between the two types of schizophrenia, which the authors believe has been achieved. Due to the complex nature of the relationship between brain and behavior, the results of neuropsychological assessment can only suggest a complex neural network dysfunction responsible for performing specific executive functions, which may be another potential limitation of this study (Alexander et al., 2012). Future projects might focus on the assessment of executive function and working memory in deficit patients, based on functional magnetic resonance imaging as well as the assessment of the consequences of impaired executive function on psychosocial functioning in deficit and NDS patients.

## Ethics Statement

This study was carried out in accordance with the recommendations of Bioethical Commission of the Pomeranian Medical University with written informed consent from all subjects. All subjects gave written informed consent in accordance with the Declaration of Helsinki. The protocol was approved by the Bioethical Commission of the Pomeranian Medical University.

## Author Contributions

All authors contributed to and have approved the final manuscript. JP-W was the principal coordinator of the grant, was involved in the study design, and took part in patient recruitment. ET managed literature searches and analyses, performed statistical analysis, wrote the first draft of the manuscript and took part patient recruitment. JS was involved in conceptualization of the project, study design, and corrected the manuscript. MM took part in patient recruitment. AS took part in patient recruitment. PB took part in patient recruitment.

## Conflict of Interest Statement

The authors declare that the research was conducted in the absence of any commercial or financial relationships that could be construed as a potential conflict of interest.
